# Association between inhaled nitric oxide treatment and long-term pulmonary function in survivors of acute respiratory distress syndrome

**DOI:** 10.1186/cc11215

**Published:** 2012-03-02

**Authors:** R Phillip Dellinger, Stephen W Trzeciak, Gerard J Criner, Janice L Zimmerman, Robert W Taylor, Helen Usansky, Joseph Young, Brahm Goldstein

**Affiliations:** 1 Division of Critical Care Medicine, Department of Medicine, Cooper University Hospital, 1 Cooper Plaza, Camden, NJ 08103, USA; 2 Section of Pulmonary and Critical Care Medicine, Department of Medicine, School of Medicine, Temple University Hospital,3401 North Broad Street, Philadelphia, PA 19140, USA; 3 Division of Critical Care Medicine, Department of Medicine, The Methodist Hospital, 6565 Fannin Street, Houston, TX 77030, USA; 4 Critical Care Medicine Department, Saint Louis University, School of Medicine, Mercy Hospital, 621 S. New Ballas Road, Saint Louis, MO 63141, USA; 5 Clinical Development Department, Ikaria Inc, 53 Frontage Road Hampton, NJ 08827-9001, USA

## Abstract

**Introduction:**

Assessment of treatments for acute respiratory distress syndrome (ARDS) has focused on short-term outcomes (for example, mortality); little information exists regarding long-term effects of ARDS treatment. Survivors of ARDS episodes may have long-term obstructive/restrictive pulmonary abnormalities and pulmonary gas exchange impairment. A 2004 prospective randomized placebo-controlled trial assessed the efficacy and safety of inhaled nitric oxide (iNO) in patients with non-septic ARDS; the primary endpoint was days alive and off assisted breathing. This analysis examined potential effects of iNO or placebo on pulmonary function six months post-treatment in ARDS survivors from that original study.

**Methods:**

ARDS survivors (*N *= 92) from a large-scale randomized, placebo-controlled study evaluating mortality after either 5 ppm iNO or placebo for up to 28 days were assessed six months post-treatment. Pulmonary function testing across seven parameters was conducted.

**Results:**

At 6 months post-treatment, results indicated significantly better absolute values for iNO versus placebo for mean ± SD total lung capacity (TLC, 5.54 ± 1.42 vs. 4.81 ± 1.00; *P *= 0.026). There were also significantly better values for mean ± SD percent predicted values for a) forced expiratory volume in 1 second (FEV_1_, 80.23 ± 21.21 vs. 69.51 ± 28.97; *P *= 0.042), b) forced vital capacity (FVC, 83.78 ± 19.37 vs. 69.84 ± 27.40; *P *= 0.019), c) FEV_1_/FVC (96.14 ± 13.79 vs. 87.92 ± 19.77; *P *= 0.033), and d) TLC (93.33 ± 18.21 vs. 76.10 ± 21.84; *P *< 0.001). Nonsignificant differences were found in absolute FEV1, FEV1/FVC, FVC, forced expiratory flow from 25% to 75% of FVC, functional residual capacity, and CO diffusion.

**Conclusions:**

ARDS patients surviving after treatment with low-dose iNO had significantly better values for select pulmonary function tests at six months post-treatment than placebo-treated patients. Further trials are warranted to determine the effects of iNO on chronic lung function in ARDS survivors, a factor in long-term morbidity and quality of life in this population.

**Trial Registration:**

A Double-blind, Randomized, Placebo-controlled, Dose-response Study of Inhaled Nitric Oxide in the Treatment of Acute Respiratory Distress Syndrome. NCT number: ISRCTN53268296

## Introduction

Inhaled nitric oxide (iNO) is a vasodilator indicated for treatment of term and near-term neonates with hypoxic respiratory failure associated with clinical or echocardiographic evidence of pulmonary hypertension. In these patients, iNO has been shown to improve oxygenation and reduce the need for extracorporeal membrane oxygenation therapy [1]. NO binds to and activates cytosolic guanylate cyclase, thereby increasing intracellular levels of cyclic guanosine 3',5'-monophosphate (cGMP). This, in turn, relaxes vascular smooth muscle, leading to vasodilatation. iNO selectively dilates the pulmonary vasculature, with minimal systemic vasculature effect as a result of efficient hemoglobin scavenging. In acute lung injury (ALI) and acute respiratory distress syndrome (ARDS), increases in partial pressure of arterial oxygen (PaO_2_) are believed to occur secondary to pulmonary vessel dilation in better-ventilated lung regions. As a result, pulmonary blood flow is redistributed away from lung regions with low ventilation/perfusion ratios toward regions with normal ratios [1-3].

The incidence of ARDS has been estimated to be approximately 75 cases per 100,000 population, although this figure is impacted by ambiguity in the causes and manifestations of ARDS [4,5]. Mortality rates in ARDS are substantial, with estimates ranging from 34% to 68% [4], highlighting the need for effective treatment.

Many pharmacologic treatments have been investigated in ARDS patients, including alprostadil [6], acetylcysteine [7], corticosteroids [8], surfactant [9], dazoxiben [10] and acyclovir [11]. All studies to date have focused on mortality as the primary endpoint. A meta-analysis of trials completed through 2004 indicated limited mortality benefit with any of the above-mentioned treatments [12].

Patients surviving an episode of ARDS may have long-term obstructive and restrictive pulmonary abnormalities as well as pulmonary gas exchange impairment [13-15]. These long-term effects may contribute to decreased quality of life (QoL), repeatedly demonstrated by ARDS survivors [13,16,17]. The importance of long-term effects following an ARDS episode has recently emerged, with clinicians noting that assessing short-term survival of ARDS is only part of its clinical impact. Therefore, treatments provided in the ICU that improve long-term ARDS outcomes (without improving immediate survival) and clinical studies examining treatment effects on later outcomes may be relevant [18].

A large-scale, randomized, blinded, placebo-controlled study carried out in the ICUs of 46 US hospitals evaluated the efficacy of low-dose (5 ppm) iNO in 385 patients with moderately severe ALI. The primary endpoint was number of days alive and off assisted breathing. Results of an intent-to-treat analysis revealed that iNO had no significant benefit versus control (nitrogen gas) as it related to mortality (23% versus 20%, respectively), days alive and off assisted breathing (mean, 10.7 versus 10.6 days), or days alive and meeting oxygenation criteria for extubation (mean, 16.7 versus 17.0 days). Treatment, however, resulted in a significant increase (*P *< 0.05) in PaO_2 _during the initial 24 hours of treatment, with improvement resolved by 48 hours [19].

Safety results for the initial 28-day study period have been reported [19] and are summarized briefly here. A total of 630 adverse events (AEs) were reported for patients treated with iNO versus 666 events for those receiving placebo. Respiratory system AEs occurred in 51% versus 61% of patients receiving iNO and placebo, respectively, primarily due to higher frequencies of pneumonia, pneumothorax, and apnea in the placebo group. Frequency of other AEs was similar in both groups [19].

The present analysis was developed *a priori *as part of the original study protocol and assessed long-term pulmonary function differences between iNO and placebo at six months post-treatment. The original rationale for long-term follow-up of pulmonary function in survivors was to assess both the safety of iNO use in ARDS and to examine any potential efficacy on the incidence of chronic lung disease in survivors. This study is the first prospective long-term analysis of pulmonary function in ARDS survivors participating in a randomized interventional clinical trial comparing iNO and placebo.

## Materials and methods

The protocol, amendments to the protocol, and local Informed Consent Forms were reviewed and approved by each of the participating hospitals' Institutional Review Board prior to initiation of patient accrual.

Inclusion and exclusion criteria and treatment details for this analysis are described elsewhere [19]; they are summarized briefly here.

### Patients

Patients had moderately severe ALI, defined by a modification of American-European Consensus Conference criteria (PaO_2_/fraction of inspired oxygen [FiO_2_] ratio of ≤ 250 mm Hg), due to causes other than severe sepsis. Patients with evidence of non-pulmonary system failure at the time of randomization and sepsis-induced ARDS were excluded. Patients were also excluded if they had sustained hypotension requiring vasopressor support, hemodynamic profiles supporting severe sepsis, severe head injury, severe burns or evidence of other significant organ system dysfunction at baseline [19].

### Treatment

Patients were randomly assigned to receive either inhaled placebo (nitrogen) or 5 ppm of iNO (INO Therapeutics Inc., Port Allen, LA, USA). Patients, healthcare professionals, and investigators were blinded to the assigned treatment. iNO was administered via INOvent^® ^delivery system (Datex-Ohmeda, Madison, WI, USA) that blended the treatment drug (nitrogen or NO at 100-ppm balance nitrogen) 1:20 with ventilator gases to achieve a target ppm value in the inspiratory limb of the ventilator [19].

All patients using the iNO delivery system received mechanical ventilatory support. Treatment continued with active or placebo gas until one of the following criteria was met: 1) end of trial (28 days); 2) death; or 3) adequate oxygenation (arterial oxygen saturation by pulse oximetry [SpO_2_] ≥ 92% or PaO_2 _of ≥ 63 mm Hg) without treatment at ventilator settings of FiO_2 _≤ 0.4 and positive end-expiratory pressure (PEEP) of ≤ 5 cm H_2_O. Decreases in treatment gas continued in 20% decrements (titrated down by 1 ppm for iNO) every 30 minutes until either the drug concentration reached 0% or oxygenation criteria were not satisfied. If oxygenation criteria were not met, drug concentration was titrated up until they were again achieved. Increments of upward titration were determined by the clinician, based on degree of arterial desaturation [19].

### Respiratory parameters measured during hospitalization

Baseline oxygenation measures included PaO_2_, arterial partial pressure of CO_2 _(PaCO_2_), SpO_2_, FiO_2_, PEEP, PaO_2_/FiO_2 _ratio, ventricular rate, tidal volume, and mean airway pressure. Respiratory parameters (FiO_2_, PEEP, and PaO_2_/FiO_2 _ratio) were recorded on case report forms every 12 hours during mechanical ventilation.

### Long-term pulmonary function measures

Pulmonary function testing (PFT) at six months post-treatment was required in both iNO- and placebo-treated patients as part of the original study design. PFTs included FEV_1_, FEV_1 _% predicted, FVC, FVC % predicted, the FEV_1_/FVC ratio, FEV_1_/FVC ratio % predicted, forced expiratory flow (FEF) from 25% to 75% of FVC (FEF_25-75%_), FEF_25-75% _% predicted, functional residual capacity (FRC), FRC % predicted, total lung capacity (TLC), TLC % predicted, CO diffusion, and CO diffusion % predicted.

### Statistical methods

All between-group differences in PFT results were evaluated using the Wilcoxon rank sum test. Between-group differences in baseline clinical and demographic characteristics were assessed with either Fisher's exact test or chi-square test for categorical variables and with Wilcoxon rank sum test for continuous variables. Baseline oxygenation and respiratory/oxygenation parameters in the two groups were compared using Wilcoxon rank sum tests. The areas under the curve (AUCs) of FiO_2 _and PEEP were calculated using the trapezoidal rule to demonstrate the total exposure of both groups during days of mechanical ventilation to supplemental oxygen and PEEP from Baseline through day 28 or day of discharge, inclusive. The null hypothesis that the respective AUCs were normally distributed was rejected employing the Shapiro-Wilk test. A Wilcoxon rank sum test was utilized to assess the differences in each median AUC between treatment groups. A *P *value < 0.05 was considered significant.

## Results

### Demographics and baseline characteristics

**Figure 1:**
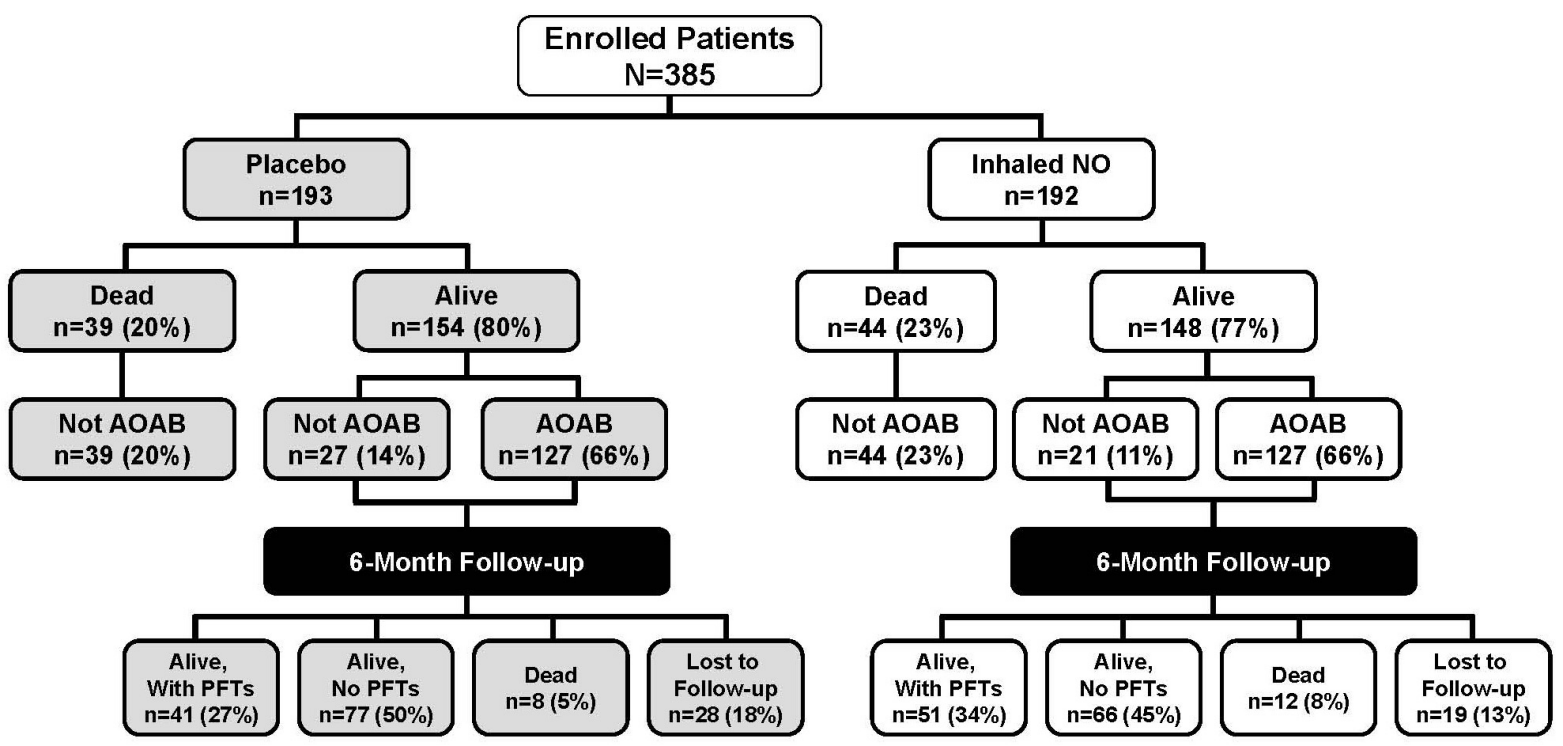
**Disposition of subjects**. AOAB, alive and off assisted breathing by day 28; NO, nitric oxide; PFT, pulmonary function test.

Final disposition of all subjects in the original study and six-month follow-up is shown in Figure 1. A total of 302 patients were survivors (alive, on or off assisted breathing) after the initial 28-day treatment period (148 in the iNO group, 154 placebo). Of these, 92 (30%) were capable of and participated in the six-month follow-up pulmonary function evaluations, 51 (55%) in the iNO group and 41 (45%) in the placebo group. The remaining surviving subjects (*n *= 210) either died prior to follow-up (*n *= 20), were lost to follow-up (*n *= 47), or did not have available PFT data (*n *= 143). Baseline patient characteristics are summarized in Table 1. The two treatment groups were well matched for all demographic variables. There were no significant differences between groups with respect to ARDS etiology, severity of illness, frequency of co-morbid chronic respiratory conditions or use of inhaled corticosteroids. More subjects had a history of tobacco use in the iNO group (26 versus 17, *P *= 0.41). There were more placebo patients who had evidence of morbid obesity (actual body weight is ≥ 35% over ideal body weight) (19 versus 13, *P *= 0.028). Regardless of the presence or absence of morbid obesity, analyses showed that the treatment effect remained the same.

**Table 1:** Baseline demographic and clinical characteristics

Parameter		Placebo	Inhaled NO	*P *Value
Age, years	N	41	51	
	Mean ± SD	47.8 ± 16.7	45.3 ± 15.3	0.494
	Range	18.4 - 84.0	16.8 - 77.9	
Sex, n (%)	Male	19 (46%)	25 (49%)	0.836
	Female	22 (54%)	26 (51%)	
Race, n (%)	Caucasian	35 (85%)	42 (82%)	0.847
	Black	4 (10%)	5 (10%)	
	Other	2 (5%)	4 (8%)	
Height, cm	N	39	51	
	Mean ± SD	168.7 ± 11.4	169.4 ± 9.2	0.912
Weight, kg	N	41	51	
	Mean ± SD	85.7 ± 24.1	76.4 ± 19.2	0.049
BW ≥ 35% IBW	N	19 (46.3%)	13 (25.5%)	0.028
Causes of ARDS,^a ^n (%)				
Pneumonia		20 (49%)	15 (29%)	0.084
Toxic gas inhalation		0 (0%)	0 (0%)	1.000
Acute pancreatitis		1 (2%)	3 (6%)	0.626
Massive blood transfusion		5 (12%)	10 (20%)	0.404
Fat emboli		1 (2%)	2 (4%)	1.000
Aspiration pneumonitis		9 (22%)	9 (18%)	0.610
Pulmonary contusion		6 (15%)	12 (24%)	0.307
Postpartum ARDS		2 (5%)	0 (0%)	0.196
Multiple trauma		14 (34%)	15 (29%)	0.657
Elective or emergency surgical procedures		9 (22%)	20 (39%)	0.114
Patient History, n (%)				
Preexisting steroid use		3 (7%)	8 (15%)	0.334
Asthma		4 (10%)	5 (10%)	1.000
COPD		6 (15%)	6 (12%)	0.761
Tobacco use		17 (41%)	26 (51%)	0.405

^a^Patients may have more than one cause of ARDS. ARDS, acute respiratory distress syndrome; BW, body weight; COPD, chronic obstructive pulmonary disorder; IBW, ideal body weight; N, number; NO, nitric oxide.

### Baseline oxygenation parameters

Baseline oxygenation parameters, including PaO_2_, PaCO_2_, SpO_2_, FiO_2_, PEEP, and PaO_2_/FiO_2 _ratio are summarized in Table 2. The patients included in this analysis were severely ill with mean baseline PaO_2_/FiO_2 _ratios of 140.5 ± 43.4 (iNO) and 136.1 ± 40.4 (placebo). Except for a clinically insignificant difference in SpO_2_, there were no significant between-group differences with respect to baseline oxygenation parameters.

**Table 2:** Baseline oxygenation and respiratory parameters

Parameter	Statistics	Placebo	Inhaled NO	*P *Value
PaO_2_, mm Hg	N	41	50	
	Mean ± SD	84.8 ± 21.4	90.6 ± 19.1	
	Median	81	86	0.068
PaCO_2_, mm Hg	N	41	50	
	Mean ± SD	39.9 ± 7.7	40.8 ± 8.4	
	Median	41	39	0.728
SpO_2_,%	N	41	50	
	Mean ± SD	95.1 ± 2.6	96.5 ± 2.6	
	Median	96	97	0.012
FiO_2_	N	41	50	
	Mean ± SD	0.65 ± 0.13	0.68 ± 0.16	
	Median	1	1	0.517
PEEP, cm H_2_O	N	41	51	
	Mean ± SD	9.5 ± 1.7	9.8 ± 2.5	
	Median	10	10	0.748
PaO_2_/FiO_2 _ratio	N	41	50	
	Mean ± SD	136.1 ± 40.4	140.5 ± 43.4	
	Median	132	130	0.774
Ventilator rate, breaths/min	N	41	50	
	Mean ± SD	14.6 ± 4.4	13.1 ± 4.2	0.069
Tidal volume, mL/kg	N	39	49	
	Mean ± SD	9.1 ± 1.7	10.3 ± 2.5	0.548
Mean airway pressure, cm H_2_O	N	37	46	
	Mean ± SD	18.3 ± 7.1	16.9 ± 5.2	0.488

FiO_2_, fraction of inspired oxygen; N, number; NO, nitric oxide; PaCO_2_, arterial pressure of CO_2_; PaO_2_, partial pressure of arterial oxygen; PEEP, positive-end expiratory pressure; SpO_2_, pulse oximetric oxygen saturation.

### Baseline respiratory parameters

Baseline respiratory parameters, including ventilator rate, tidal volume, and mean airway pressure are also summarized in Table 2. There were no significant differences between groups for any of these measures.

### Respiratory parameters during mechanical ventilation

**Figure 2:**
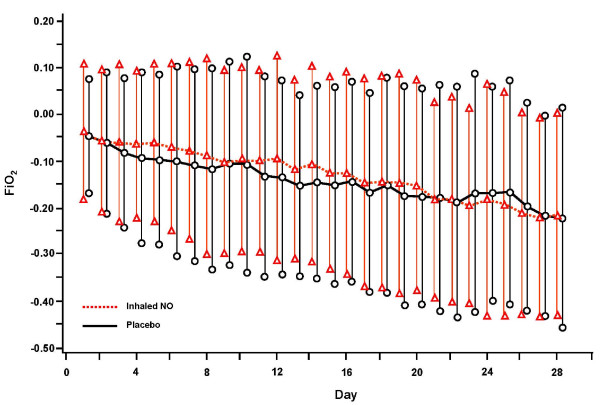
**Aggregate of individual subject data of daily change (mean ± SD) from baseline parameter for FiO_2 _through day 28; FiO_2_, fraction of inspired oxygen; NO, nitric oxide**.

**Figure 3:**
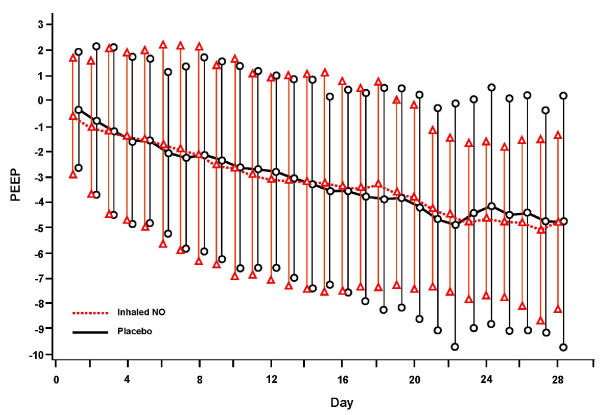
**Aggregate of individual subject data of daily change (mean ± SD) from baseline parameter for PEEP through day 28**. NO, nitric oxide; PEEP, positive-end expiratory pressure.

**Figure 4:**
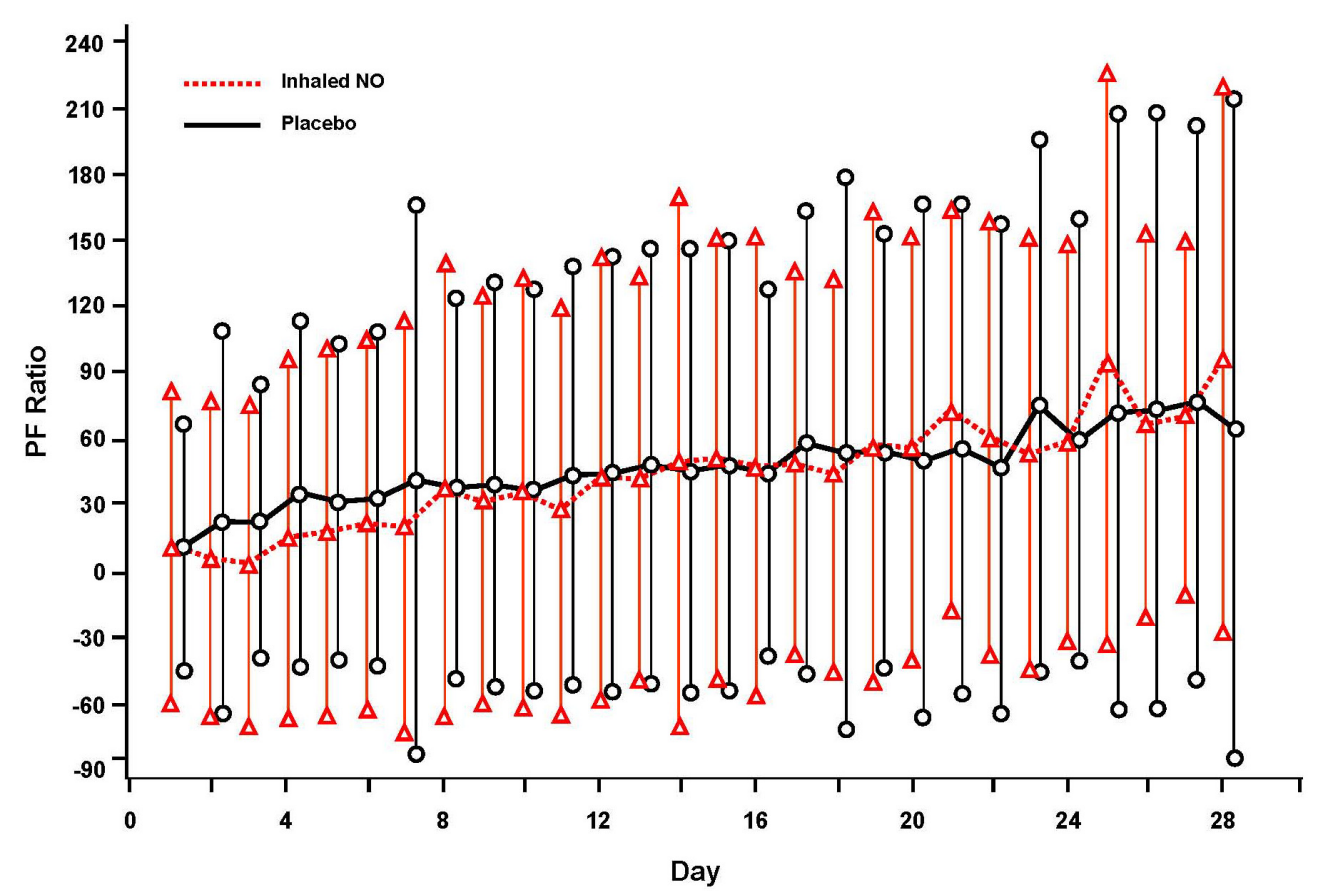
**Aggregate of individual subject data of daily change (mean ± SD) from baseline parameter for P/F ratio through day 28**. NO, nitric oxide; PF, PaO_2_/FiO_2 _ratio.

There were no significant differences between groups for aggregate daily per-patient changes from baseline parameters in supplemental oxygen, PEEP, or PaO_2_/FiO_2 _ratio for patients receiving mechanical ventilation (Figures 2, 3 and 4).

However, when calculating the duration of exposure (AUC) over the length of mechanical ventilation for total FiO_2 _(6.3 ± 4.5 versus 7.6 ± 4.7 (%days) for iNO and placebo groups, respectively; *P *= 0.151) and total PEEP (96.3 ± 75.9 versus 113.4 ± 81.1 (mmHgdays), *P *= 0.261), although not statistically significant, the iNO group did have less cumulative exposure to both variables (Table 3).

**Table 3:** Duration of exposure parameters (AUC) during mechanical ventilation from baseline through day 28 or discharge

AUC Parameter	Placebo (N = 41)	Inhaled NO (N = 51)	*P *Value
Inhaled NO (ppm days)^a^	0	114 ± 102	NA
FiO_2 _(% days)	7.6 ± 4.7	6.3 ± 4.5	0.151
PEEP (mmHg days)	113.4 ± 81.1	96.3 ± 75.9	0.261

^a^Values are mean ± SD unless otherwise indicated. AUC, area under the curve; FiO_2_, fraction of inspired oxygen; N, number; NO, nitric oxide; PaO_2_, partial pressure of arterial oxygen; PEEP, positive-end expiratory pressure.

### Pulmonary function tests at six months

**Figure 5:**
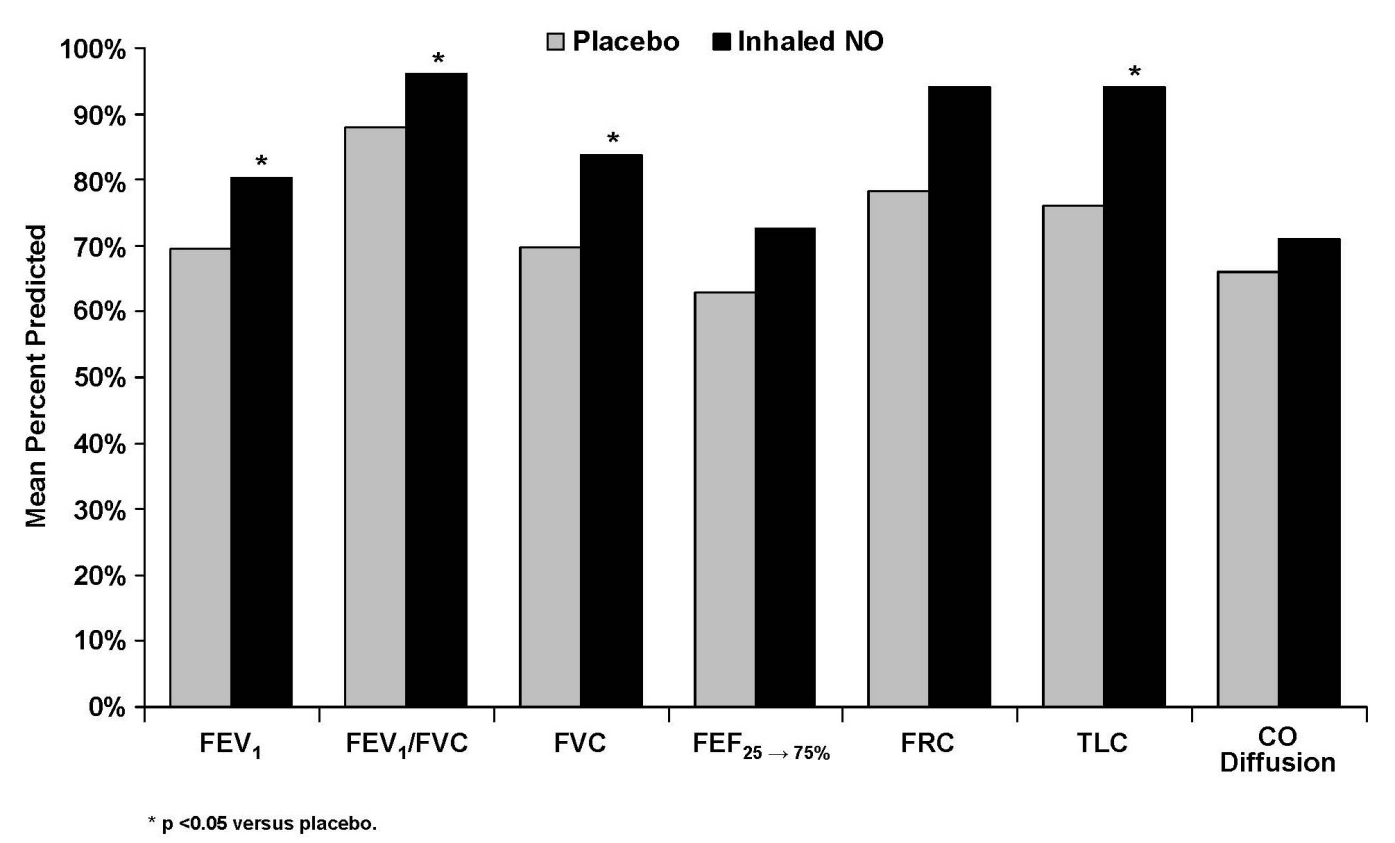
**Mean percent predicted pulmonary function comparisons between placebo and inhaled nitric oxide**. NO, nitric oxide.

Results for PFTs at six months post-treatment with placebo or iNO are summarized in Table 4 and presented as comparison percent between treatment groups in Figure 5. Study results indicated significantly better values for patients treated with iNO versus placebo for FEV_1 _% predicted (*P *= 0.042), FVC % predicted (*P *= 0.019), FEV_1_/FVC % predicted (*P *= 0.033), TLC (*P *= 0.026), and TLC % predicted (*P *< 0.001). No significant differences were observed in FEV_1_, FEV_1_/FVC, FVC, FEF_25-75%_, FRC, or CO diffusion values, nor for percent predicted values for FEF_25-75%_, FRC, and CO diffusion.

**Table 4:** Pulmonary function test results at six months

Parameter	Statistics	Placebo	Inhaled NO	*P *Value
FEV_1_, L	N	41	51	
	Mean ± SD	2.29 ± 0.71	2.64 ± 0.91	0.1161
FEV_1_, % predicted	N	41	50	
	Mean ± SD	69.51 ± 28.97	80.23 ± 21.21	0.042
FEV_1_/FVC, %	N	40	51	
	Mean ± SD	72.89 ± 20.20	77.45 ± 15.19	0.155
FEV_1_/FVC, % predicted	N	37	49	
	Mean ± SD	87.92 ± 19.77	96.14 ± 13.79	0.033
FVC, L	N	41	51	
	Mean ± SD	3.01 ± 0.96	3.36 ± 1.09	0.163
FVC, % predicted	N	41	50	
	Mean ± SD	69.84 ± 27.40	83.78 ± 19.38	0.019
FEF_25-75%_, L/sec	N	41	51	
	Mean ± SD	12.25 ± 55.86	26.34 ± 84.50	0.121
FEF_25-75%_, % predicted	N	41	50	
	Mean ± SD	62.96 ± 36.26	72.50 ± 27.71	0.154
FRC, L	N	33	44	
	Mean ± SD	2.64 ± 0.71	3.00 ± 0.94	0.113
FRC, % predicted	N	32	43	
	Mean ± SD	78.19 ± 29.95	93.98 ± 25.55	0.109
TLC, L	N	32	44	
	Mean ± SD	4.81 ± 1.00	5.54 ± 1.42	0.026
TLC, % predicted	N	31	43	
	Mean ± SD	76.10 ± 21.84	93.33 ± 18.21	< 0.001
CO diffusion, mL/min/mm Hg	N	33	42	
	Mean ± SD	17.87 ± 6.37	18.25 ± 6.77	0.709
CO diffusion, % predicted	N	32	42	
	Mean ± SD	65.96 ± 23.23	71.02 ± 20.79	0.492

FEF, forced expiratory flow; FEV_1_, forced expiratory volume in 1 second; FRC, functional residual capacity; FVC, forced vital capacity; N, number; NO, nitric oxide; TLC, total lung capacity.

### Baseline characteristics of survivors and patients with no follow-up at six months

Table 5 shows that the two treatment groups were well matched for all demographic variables except for a slight but statistically significant difference in age and the incidence of other types of preexisting lung disease with the patients without six month follow-up data being slightly older and having a greater incidence of preexisting lung disease of 'other' etiologies.

**Table 5:** Comparison of baseline demographic and clinical characteristics between survivors and patients without six month follow-up

Parameter		Survivors	No 6-Month Follow-up	*P*-Value
Age (years)	N	92	293	
	Mean ± SD	46.4 ± 15.9	50.9 ± 17.4	0.044
	Range	16.8 - 84.0	17.6 - 87.0	
Sex, n (%)	Male	44 (47.8%)	161 (54.9%)	0.281
	Female	48 (52.2%)	132 (45.1%)	
Race, n (%)	Caucasian	77 (83.7%)	227 (77.5%)	0.423
	Black	9 (9.8%)	44 (15.0%)	
	Other	6 (6.5%)	22 (7.5%)	
Height (cm)	N	90	277	
	Mean ± SD	169.1 ± 10.2	169.2 ± 11.6	0.856
Weight (kg)	N	92	292	
	Mean ± SD	80.5 ± 21.9	79.4 ± 20.1	0.840
Causes of ARDS,^a ^n (%)				
Pneumonia		35 (38.0%)	142 (48.5%)	0.093
Toxic Gas Inhalation		0 (0%)	0 (0%)	1.000
Acute Pancreatitis		4 (4.3%)	12 (4.1%)	1.000
Masssive Blood Transfusion		15 (16.3%)	31 (10.6%)	0.144
Fat Emboli		3 (3.3%)	7 (2.4%)	0.708
Aspiration Pneumonitis		18 (19.6%)	72 (24.6%)	0.397
Pulmonary Contusion		18 (19.6%)	51 (17.4%)	0.642
Post-Partum ARDS		2 (2.2%)	1 (0.3%)	0.143
Multiple Trauma		29 (31.5%)	74 (25.3%)	0.280
Elective or Emergency Surgical Procedures		29 (31.5%)	100 (34.1%)	0.705
Patient History, n (%)				
Preexisting steroid use		11 (12.0%)	34 (11.6%)	1.000
Asthma		9 (9.8%)	30 (10.2%)	1.000
COPD		12 (13.0%)	36 (12.3%)	0.857
Tobacco use		47 (51.1%)	121 (41.3%)	0.117
Other lung disease^b^		0 (0%)	18 (6.1%)	0.009

^a^Patients may have more than one cause of ARDS. ^b^Patients may have more than one preexisting disease including: cancer, bronchitis, amiodarone toxicity, and status/post lung resection. ARDS, acute respiratory distress; COPD, chronic obstructive pulmonary disorder; N, number; NO, nitric oxide.

## Discussion

Clinical trials evaluating numerous interventions have repeatedly failed to demonstrate significant benefit in decreasing mortality in ARDS patients [12,20]. Endpoints such as long-term morbidity, or a shift of focus to short- and long-term respiratory changes in survivors of ARDS, may be important when evaluating established and emerging ARDS treatments. In a study evaluating 50 long-term ARDS survivors, assessed a median of 5.5 years after ICU discharge, 54% had impairment (defined as < 80% predicted value) in at least one pulmonary function measure, including decreases in FEV_1_/FVC ratio consistent with airflow obstruction in 16 (32%), residual volume in 14 (28%), TLC in 10 (20%), and diffusing capacity in 8 (16%) patients. Seven patients (14%) had multiple pulmonary function abnormalities. Overall, ARDS survivors described a 25% reduction in physical and physical role function compared with age- and sex-matched controls (*P *< 0.001). In addition, those with more than one pulmonary function abnormality had significantly decreased health-related QoL parameters (that is, general/mental health, physical/social function, and vitality) compared with those having one or no abnormalities [21].

While part of a larger study examining the short-term (28-day) effects of iNO on mortality and need for assisted breathing, this six-month follow-up assessment is the first prospective analysis evaluating iNO effects on long-term pulmonary function in ARDS survivors. The original clinical trial [19], as well as a meta-analysis of 12 randomized controlled trials in ALI or ARDS patients [20], indicated no significant benefit of iNO in decreasing mortality and only transient effects on physiological endpoints, such as PaO_2_/FiO_2 _ratio. The results of this six-month follow-up indicate an association between iNO and better PFT results at six months post-treatment. The patients receiving iNO had less restrictive defect as reflected by higher FEV% predicted, FVC % predicted and TLC. This association of iNO treatment with reduced restrictive defect was not accompanied by a significantly higher CO diffusion and raises the question of potential systemic effect of iNO on muscle function. The effects of iNO on systemic tissue beds is a rapidly expanding, but only recently recognized, area of research discovery.

The clinical significance of longer-term lung function and QoL in ARDS survivors has been examined. Although not linked in all studies, results from one long-term follow-up of ARDS survivors indicated that both FEV_1 _and FVC at 12 months post-episode were correlated with the physical function domain of two validated QoL questionnaires [14].

Additionally, the cumulated aggregate per-subject values for FiO_2_, PEEP, and PaO_2_/FiO_2 _exposure days, while not reaching statistical significance, were less in the iNO-treated patients compared with those in the group treated with placebo. Taken together, the differences in six-month PFTs, as well as the aggregate oxygenation exposure data, suggest a potential long-term physiologic effect of iNO on lung function in ARDS survivors.

There was an increased incidence of morbid obesity in placebo patients in the study. As patients with morbid obesity have an increased risk for pulmonary complications, this could be hypothesized to partially explain some of the results. Regardless of the presence or absence of morbid obesity, analyses showed that the treatment effect remained the same (Additional files 1, 2, 3 and 4). Thus, the effects seen at six months are not explained by the imbalance in number of patients with morbid obesity between placebo and treatment groups.

iNO exerts its physiologic effects via cGMP-mediated relaxation of the vascular smooth muscle and selective dilation of the pulmonary vasculature. In addition, several other potential mechanisms may underlie better performance of iNO on pulmonary function. ARDS is associated with pronounced elevations in multiple inflammatory markers [22,23] and several studies have suggested that these may be attenuated by iNO. Studies with experimental animals [24] and ARDS patients [25] have shown that iNO significantly decreases pulmonary concentrations of IL-8 and neutrophils, as well as significantly inhibiting the formation of platelet-leukocyte aggregates (an effect correlated with an NO-dependent inhibition of platelet P-selectin expression). This may potentially lead to improvement of microcirculation in vascular beds, including muscle [26]. In another study with ARDS patients, iNO was found to significantly decrease H_2_O_2 _production and β_2_-integrin CD11b/CD18 expression by polymorphonuclear leukocytes. In addition, iNO decreased IL-6 and IL-8 concentrations in bronchoalveolar lavage (BAL) fluid [27]. The well-known mechanism of action for iNO (for example, cGMP-mediated vasodilatation) likely explains the decreased duration of FiO_2 _and PEEP exposure in the original 28-day trial; the aforementioned insight into other mechanisms of iNO in ARDS patients offers additional focus for research regarding long-term effects in ARDS survivors.

In the original study, iNO did not improve short-term mortality in patients with ARDS, despite transient physiologic benefit [19]. With ARDS patients in general, 26% to 44% of deaths typically occur within 72 hours of ARDS onset; these deaths are more often attributable to events such as sepsis with multiple organ failure (30% to 50%) than to respiratory failure (13% to 19%) [28]. In addition, changes in oxygenation sustained for only 24 hours with iNO have been shown to be insufficient to alter mortality in patients with ARDS/ALI [20]. Given these data, short-term outcomes with treatments such as iNO may not prove clinically impactful; however, the longer-term impact of iNO treatment in ARDS survivors, based on the data herein and the potential mechanisms of iNO in ARDS patients, warrant additional investigation.

### Limitations

This analysis had limitations, primarily: 1) the large percentage of subjects lost to follow-up who did not have PFTs performed at six months; and 2) the inability to obtain premorbid PFTs. Even though the former constituted a protocol violation, the reasons for this occurring are not available, potentially influencing the results via significant bias or confounding. Additionally, while the lack of premorbid PFTs would have provided valuable insight, from a practical study standpoint, obtaining these values was not possible. The fact that the baseline characteristics between groups were very similar, especially with respect to severity of illness, co-morbid chronic respiratory conditions and use of inhaled corticosteroids, suggests that these potential influences may have been minimized.

There was a small but statistically significant difference in baseline SpO_2 _that favored the iNO group. Tidal volume was higher, and ventilator rate and mean airway pressure were lower in patients receiving iNO; however, there was no consistent pattern of these small, non-significant differences that would support an influence on pulmonary function at the six-month follow-up. Finally, the inclusion criteria of the original study did not exclude preexisting lung disease and treatment assignment was not stratified on that basis [19].

## Conclusions

While current clinical research regarding ARDS treatment has focused on mortality and short-term effects of treatment, it is important to consider chronic lung effects in ARDS survivors, which could be a cause of long-term morbidity and reduction of QoL in this population. Results from this six-month analysis show that ARDS survivors who received iNO had significantly better PFT parameters versus those who received placebo, as indicated by decreased restrictive defect.

These results support consideration of further clinical trials to determine the longer-term effects of iNO on the incidence and severity of chronic lung disease in ARDS patients. Additional outcomes that should be explored include measures of health-related QoL, healthcare utilization, and overall patient management cost.

## Key messages

1. Multiple clinical trials have failed to show a survival benefit of inhaled nitric oxide (INO) in ARDS.

2. Inhaled nitric oxide has known actions that might be associated with long term improvement in pulmonary function

3. Long term functional outcome differences might be present for acute interventions in ARDS that target 28 day mortality even if primary outcome endpoint is not met.

4. In a large randomized clinical trial that failed to demonstrate survival benefits when ARDS was treated with INO, PFTs performed at 180 days in a survivor sample demonstrated less restrictive defect in survivors given INO.

5. Additional studies are needed to prove or disprove this secondary endpoint analysis.

## Abbreviations

AE: adverse event; ALI: acute lung injury; ARDS: acute respiratory distress syndrome; AUC: area under the curve; cGMP: cyclic guanosine 3',5'-monophosphate; FEF: forced expiratory flow; FEF_25-75%_: forced expiratory flow from 25% to 75% of forced vital capacity; FEV_1_: forced expiratory volume in 1 second; FiO_2_: fraction of inspired oxygen; FRC: functional residual capacity; FVC: forced vital capacity; IL: interleukin; iNO: inhaled nitric oxide; PaCO_2_: arterial partial pressure of CO_2_; PaO_2_: partial pressure of arterial oxygen; PEEP: positive end-expiratory pressure; PFT: pulmonary function testing; QoL: quality of life; SpO_2_: arterial oxygen saturation by pulse oximetry; TLC: total lung capacity.

## Competing interests

Cooper University Hospital has been, in the past, reimbursed by Ikaria for consulting time by R. Phillip Dellinger. Robert W. Taylor, Janice L. Zimmerman, and Gerard J. Criner have no financial disclosure. Stephen Trzeciak receives material support for an ongoing clinical trial of inhaled nitric oxide from Ikaria, Inc. Mr. Young, Dr Usansky (former) and Dr. Goldstein are employees of Ikaria, Inc. Mr. Young and Dr Goldstein have stock in that company. This study was sponsored by INO Therapeutics LLC (formerly Ohmeda PPD, Inc.). Editorial support for this article was provided by Albert M. Balkiewicz, MSc, of Peloton Advantage, LLC and was funded by INO Therapeutics LLC, a subsidiary of Ikaria, Inc. Statistical analysis was provided by Ikaria, Inc.

## Authors' contributions

GC, PD, RT, JZ and JY participated in the study concept and design. GC and RT participated in the acquisition of data. JY performed the statistical analysis. GC, RP, BG, RT, ST, HU and JY participated in the analysis and interpretation of the data. PD, BG and JY participated in drafting the manuscript. All authors participated in the critical revision of the manuscript for important intellectual content and read and approved the final version.

## Supplementary Material

Additional file 1 **Pulmonary function test results at six months in subjects with morbid obesity**. Demonstrates full pulmonary function studies in enrolled patients who were morbidly obese at time of enrollment. Click here for file

Additional file 2 **Pulmonary function test results at six months in subjects without morbid obesity**. Demonstrates full pulmonary function studies in enrolled patients who were not morbidly obese at time of enrollment. Click here for file

Additional file 3 **Obesity effect on pulmonary function test results at six months in subjects treated with placebo**. This data demonstrate the effect of obesity on pulmonary function tests performed six months from the enrollment in the study who were treated with placebo. Click here for file

Additional file 4 **Obesity effect on pulmonary function test results at six months in subjects treated with INO**. This data demonstrate the effect of obesity on pulmonary function tests performed 6 months from the enrollment in the study who were treated with inhaled nitric oxide. Click here for file
