# Supplementary material for: Association between inhaled nitric oxide treatment and long-term pulmonary function in survivors of acute respiratory distress syndrome
Source: Crit Care. 2012 Mar 2;16(2):R36. doi: 10.1186/cc11215 (PMC3681348; doi:10.1186/cc11215)
Supplement: Additional file 1 — Pulmonary function test results at six months in subjects with morbid obesity. Demonstrates full pulmonary function studies in enrolled patients who were morbidly obese at time of enrollment. [file cc11215-S1.RTF]

Additional File 1
Pulmonary Function Test Results at 6 Months
Subjects with Morbid Obesity

Parameter	Statistics	Placebo	Inhaled NO	P-Value	
FEV1, L	N	19	13		
	Mean ± SD	2.17 ± 0.69	2.70 ± 1.16	0.258	
FEV1, % predicted	N	19	13		
	Mean ± SD	64.72 ± 28.03	81.85 ± 22.19	0.173	
FEV1/FVC, %	N	19	13		
	Mean ± SD	74.79 ± 10.20	78.85 ± 9.75	0.111	
FEV1/FVC, % predicted	N	16	12		
	Mean ± SD	85.25 ± 9.09	94.75 ± 13.66	0.074	
FVC, L	N	19	13		
	Mean ± SD	2.92 ± 0.84	3.42 ± 1.32	0.309	
FVC, % predicted	N	19	13		
	Mean ± SD	68.95 ± 28.66	82.77 ± 21.69	0.249	
FEF25-75%, L/sec	N	17	13		
	Mean ± SD	1.91 ± 1.06	2.72 ± 1.60	0.160	
FEF25-75%, % predicted	N	19	13		
	Mean ± SD	52.33 ± 26.40	75.92 ± 30.90	0.040	
FRC, L	N	14	9		
	Mean ± SD	2.46 ± 0.77	3.06 ± 1.18	0.175	
FRC, % predicted	N	14	9		
	Mean ± SD	69.48 ± 33.46	96.44 ± 33.50	0.196	
TLC, L	N	13	9		
	Mean ± SD	4.87 ± 0.88	5.96 ± 2.21	0.205	
TLC, % predicted	N	13	9		
	Mean ± SD	74.57 ± 24.03	97.22 ± 29.84	0.161	
CO diffusion, ml/min/mm Hg	N	14	8		
	Mean ± SD	19.81 ± 7.74	21.11 ± 11.22	0.865	
CO diffusion, % predicted	N	14	8		
	Mean ± SD	64.17 ± 23.67	74.13 ± 27.53	0.585	


FEF = forced expiratory flow; FEV1 = forced expiratory volume in 1 second; FRC = functional residual capacity;
FVC = forced vital capacity; NO = nitric oxide; TLC = total lung capacity.
