# Supplementary material for: Association between inhaled nitric oxide treatment and long-term pulmonary function in survivors of acute respiratory distress syndrome
Source: Crit Care. 2012 Mar 2;16(2):R36. doi: 10.1186/cc11215 (PMC3681348; doi:10.1186/cc11215)
Supplement: Additional file 2 — Pulmonary function test results at six months in subjects without morbid obesity. Demonstrates full pulmonary function studies in enrolled patients who were not morbidly obese at time of enrollment. [file cc11215-S2.RTF]

Additional File 2
Pulmonary Function Test Results at 6 Months
Subjects without Morbid Obesity

Parameter	Statistics	Placebo	Inhaled NO	P-Value	
FEV1, L	N	20	38		
	Mean ± SD	2.44 ± 0.70	2.62 ± 0.82	0.595	
FEV1, % predicted	N	20	37		
	Mean ± SD	77.42 ± 26.47	79.66 ± 21.14	0.380	
FEV1/FVC, %	N	19	38		
	Mean ± SD	74.94 ± 21.94	76.98 ± 16.73	0.892	
FEV1/FVC, % predicted	N	19	37		
	Mean ± SD	90.79 ± 26.25	96.59 ± 13.99	0.622	
FVC, L	N	20	38		
	Mean ± SD	3.15 ± 1.06	3.35 ± 1.01	0.462	
FVC, % predicted	N	20	37		
	Mean ± SD	73.67 ± 23.41	84.14 ± 18.81	0.120	
FEF25-75%, L/sec	N	20	33		
	Mean ± SD	2.70 ± 1.41	2.58 ± 1.26	0.762	
FEF25-75%, % predicted	N	20	37		
	Mean ± SD	76.96 ± 39.71	71.30 ± 26.85	0.525	
FRC, L	N	17	35		
	Mean ± SD	2.80 ± 0.59	2.99 ± 0.89	0.619	
FRC, % predicted	N	17	34		
	Mean ± SD	86.27 ± 26.03	93.32 ± 23.59	0.726	
TLC, L	N	17	35		
	Mean ± SD	4.87 ± 1.02	5.43 ± 1.16	0.128	
TLC, % predicted	N	17	34		
	Mean ± SD	78.11 ± 21.03	92.29 ± 14.18	0.007	
CO diffusion, ml/min/mm Hg	N	17	34		
	Mean ± SD	16.90 ± 4.78	17.58 ± 5.27	0.556	
CO diffusion, % predicted	N	17	34		
	Mean ± SD	68.49 ± 23.68	70.29 ± 19.32	0.881	


FEF = forced expiratory flow; FEV1 = forced expiratory volume in 1 second; FRC = functional residual capacity;
FVC = forced vital capacity; NO = nitric oxide; TLC = total lung capacity.
