# Supplementary material for: Association between inhaled nitric oxide treatment and long-term pulmonary function in survivors of acute respiratory distress syndrome
Source: Crit Care. 2012 Mar 2;16(2):R36. doi: 10.1186/cc11215 (PMC3681348; doi:10.1186/cc11215)
Supplement: Additional file 3 — Obesity effect on pulmonary function test results at six months in subjects treated with placebo. This data demonstrate the effect of obesity on pulmonary function tests performed six months from the enrollment in the study who were treated with placebo. [file cc11215-S3.RTF]

Additional File 3
Obesity Effect on Pulmonary Function Test Results at 6 Months
Subjects treated with Placebo

Parameter	Statistics	Morbid
Obesity	Not Morbid
Obesity	P-Value	
FEV1, L	N	19	20		
	Mean ± SD	2.17 ± 0.69	2.44 ± 0.70	0.339	
FEV1, % predicted	N	19	20		
	Mean ± SD	64.72 ± 28.03	77.42 ± 26.47	0.160	
FEV1/FVC, %	N	19	19		
	Mean ± SD	74.79 ± 10.20	74.94 ± 21.94	0.492	
FEV1/FVC, % predicted	N	16	19		
	Mean ± SD	85.25 ± 9.09	90.79 ± 26.25	0.091	
FVC, L	N	19	20		
	Mean ± SD	2.92 ± 0.84	3.15 ± 1.06	0.747	
FVC, % predicted	N	19	20		
	Mean ± SD	68.95 ± 28.66	73.67 ± 23.41	0.789	
FEF25-75%, L/sec	N	17	20		
	Mean ± SD	1.91 ± 1.06	2.70 ± 1.41	0.067	
FEF25-75%, % predicted	N	19	20		
	Mean ± SD	52.33 ± 26.40	76.96 ± 39.71	0.052	
FRC, L	N	14	17		
	Mean ± SD	2.46 ± 0.77	2.80 ± 0.59	0.137	
FRC, % predicted	N	14	17		
	Mean ± SD	69.48 ± 33.46	86.27 ± 26.03	0.040	
TLC, L	N	13	17		
	Mean ± SD	4.87 ± 0.88	4.87 ± 1.02	0.933	
TLC, % predicted	N	13	17		
	Mean ± SD	74.57 ± 24.03	78.11 ± 21.03	0.675	
CO diffusion, ml/min/mm Hg	N	14	17		
	Mean ± SD	19.81 ± 7.74	16.90 ± 4.78	0.302	
CO diffusion, % predicted	N	14	17		
	Mean ± SD	64.17 ± 23.67	68.49 ± 23.68	0.648	


FEF = forced expiratory flow; FEV1 = forced expiratory volume in 1 second; FRC = functional residual capacity;
FVC = forced vital capacity; NO = nitric oxide; TLC = total lung capacity.
