# Supplementary material for: Association between inhaled nitric oxide treatment and long-term pulmonary function in survivors of acute respiratory distress syndrome
Source: Crit Care. 2012 Mar 2;16(2):R36. doi: 10.1186/cc11215 (PMC3681348; doi:10.1186/cc11215)
Supplement: Additional file 4 — Obesity effect on pulmonary function test results at six months in subjects treated with INO. This data demonstrate the effect of obesity on pulmonary function tests performed 6 months from the enrollment in the study who were treated with inhaled nitric oxide. [file cc11215-S4.RTF]

Additional File 4
Obesity Effect on Pulmonary Function Test Results at 6 Months
Subjects treated with INO

Parameter	Statistics	Morbid
Obesity	Not Morbid
Obesity	P-Value	
FEV1, L	N	13	38		
	Mean ± SD	2.70 ± 1.16	2.62 ± 0.82	0.871	
FEV1, % predicted	N	13	37		
	Mean ± SD	81.85 ± 22.19	79.66 ± 21.14	0.842	
FEV1/FVC, %	N	13	38		
	Mean ± SD	78.85 ± 9.75	76.98 ± 16.73	0.721	
FEV1/FVC, % predicted	N	12	37		
	Mean ± SD	94.75 ± 13.66	96.59 ± 13.99	0.701	
FVC, L	N	13	38		
	Mean ± SD	3.42 ± 1.32	3.35 ± 1.01	0.923	
FVC, % predicted	N	13	37		
	Mean ± SD	82.77 ± 21.69	84.14 ± 18.81	0.757	
FEF25-75%, L/sec	N	13	33		
	Mean ± SD	2.72 ± 1.60	2.58 ± 1.26	0.903	
FEF25-75%, % predicted	N	13	37		
	Mean ± SD	75.92 ± 30.90	71.30 ± 26.85	0.690	
FRC, L	N	9	35		
	Mean ± SD	3.06 ± 1.18	2.99 ± 0.89	0.930	
FRC, % predicted	N	9	34		
	Mean ± SD	96.44 ± 33.50	93.32 ± 23.59	0.754	
TLC, L	N	9	35		
	Mean ± SD	5.96 ± 2.21	5.43 ± 1.16	0.494	
TLC, % predicted	N	9	34		
	Mean ± SD	97.22 ± 29.84	92.29 ± 14.18	0.881	
CO diffusion, ml/min/mm Hg	N	8	34		
	Mean ± SD	21.11 ± 11.22	17.58 ± 5.27	0.501	
CO diffusion, % predicted	N	8	34		
	Mean ± SD	74.13 ± 27.53	70.29 ± 19.32	0.949	


FEF = forced expiratory flow; FEV1 = forced expiratory volume in 1 second; FRC = functional residual capacity;
FVC = forced vital capacity; NO = nitric oxide; TLC = total lung capacity.
